# Confidence and use of physical examination and point-of-care ultrasonography for detection of abdominal or pleural free fluid. A cross-sectional survey

**DOI:** 10.1007/s11739-021-02781-1

**Published:** 2021-06-20

**Authors:** Antonio Leidi, Antoine Saudan, Guillaume Soret, Frédéric Rouyer, Christophe Marti, Jérôme Stirnemann, Jean-Luc Reny, Olivier Grosgurin

**Affiliations:** 1 General Internal Medicine, Department of Medicine, Geneva University Hospitals, Geneva, Switzerland; 2 Faculty of Medicine, Geneva University, Geneva, Switzerland; 3 Emergency Medicine, Department of Acute Medicine, Geneva University Hospitals, Geneva, Switzerland

**Keywords:** Physical examination, POCUS, Ultrasonography, Free fluid, Ascites, Pleural effusion

## Abstract

Physical examination (PE) has always been a corner stone of medical practice. The recent advances in imaging and fading of doctors’ ability in performing it, however, raised doubts on PE usefulness. Point-of-care ultrasonography (POCUS) is gaining ground in medicine with the detection of free fluids being one of its main applications. To estimate physicians’ confidence and use of PE and POCUS for the detection of abdominal or pleural free fluid, we conducted a cross-sectional survey. In all, 246 internal and emergency medicine physicians answered to the survey (197 in-hospital physicians and 49 general practitioners; response rate 28.5%). Almost all declared to perform PE in case of suspected ascites or pleural effusion (88% and 90%, respectively). The highest rates of confidence were observed in conventional PE signs (91% for diminished breath sounds, 80% for dullness to thorax percussion, and 66% for abdominal flank dullness). For the remaining signs, rates of confidence were less than 53%. Physicians with > 15 years of experience and POCUS-naïve doctors reported higher confidence in PE. Most of emergency and almost half of internal medicine physicians (78% and 44%, respectively) attended a structured POCUS course. POCUS use was higher among trained physicians for both ascites (84% vs 50%, *p* < 0.001) and pleural effusion (80% vs 34%, *p* < 0.001). Similarly, higher POCUS use was observed in younger physicians. In conclusion, PE is frequently performed and rates of confidence are low for most PE signs, especially among young doctors and POCUS users. This detailed inventory suggests an ongoing shift towards POCUS integration in clinical practice.

## Introduction

Physical examination (PE), historically considered as an essential component of medical practice and part of the identity of physicians [1], is nowadays highly debated. Several reasons have been advanced, going from technological advances in imaging to loss of physicians’ skills in PE practice with succeeding generations, and difficulties of society to face incertitude [2, 3]. If on one hand, PE is still considered crucial for doctor–patient human relationship [4], on the other, diagnostic performances are often unsatisfactory [5, 6]. Little teaching time is dedicated to PE in faculties and an alarming lack of competence has been highlighted by studies among medical students and residents [7, 8]. The detection of abdominal and pleural free fluids is of uttermost importance for diagnostic and therapeutic purposes. Several physical signs have been described for ascites and pleural effusion; most of them lack sensitivity and/or specificity [9–12]. On the contrary, ultrasonography (US) is considered as the *gold standard* for ascites and pleural effusion detection; it can reveal fluid volumes of less than 100 ml and 20 ml in the peritoneal cavity and the pleura, respectively [12–14]. Even in unexperienced hands, US is more accurate than physical examination and chest X-ray [13]. Moreover, it can guide free fluid punctures raising the success rate [15] and reducing complications [16] when compared with the traditional landmark technique. Point-of-care ultrasonography (POCUS) is characterised by the bedside use of US by the front-line physician to answer focused clinical questions, guide procedures, and monitor therapies [17]. POCUS is integrated in the primary clinical evaluation along with history taking and PE [18]. The use and availability of this technology is in constant expansion in hospital and ambulatory medicine [19]. POCUS has been endorsed by international societies of internal and emergency medicine [17, 20, 21] and integrated in pre-graduate medical curricula [22, 23]. The detection of abdominal and pleural free fluid with POCUS is currently part of the Swiss medical school pre-graduate learning objectives [24].

If the use and perceived usefulness of general PE was generally high in preceding surveys [25, 26], no study specifically targeted the question to abdominal and pleural free fluid recognition nor did it in relationship with POCUS use. The aim of the present study was to estimate the use of PE and POCUS in patients with suspected ascites or pleural effusion among general practitioners (GP), internal medicine (IM) and emergency medicine (EM) physicians, working in French-speaking part of Switzerland. In addition, self-reported confidence in PE signs was estimated.

## Methods

A web-based anonymous survey was designed to answer the following four questions: (1) use of PE (any sign) in case of suspected abdominal or pleural free fluid, (2) confidence in 4 signs of ascites (flank dullness, shifting dullness, fluid wave and ‘glaçon’ sign) and 5 signs of pleural effusion (dullness to percussion, asymmetric chest expansion, reduced tactile vocal fremitus, diminished breath sounds, pleural friction rub), (3) use of POCUS and (4) frequency of radiologist referral for diagnosis.

Participants replied using a 5-point Likert scale if they used (1 = “Strongly agree” to 3 = “Neutral” to 5 = “Strongly disagree”) the previously reported PE signs in their common practice and how frequent (1 = “Never” to 3 = “Five to ten times” to 5 = “More than twenty times”) they had been using them in last three months. They additionally reported how confident (1 = “Very Confident” to 3 = “Neutral” to 5 = “Completely unconfident”) they were in these signs. For purposes of simplicity, answers on PE use were grouped in a dichotomic way in “Use” (i.e. “Strongly agree” to “Agree”) and “Don’t use” (i.e. “Neutral” to “Strongly disagree”). Answer on frequency were grouped in “Often” (i.e. “Five to ten times” to “More than twenty times”) and “Seldom” (i.e. “Never” or “Less than five times”) and answers on confidence in “Confident” (i. e. “Very confident” and “Confident”) and “Unconfident” (i.e. “Neutral” to “Completely unconfident”). To estimate the global confidence in PE, a 1 to 6 score was attributed to each PE sign as follows: “Very confident” = 1, “Confident” = 2, “Neutral” = 3, “Unconfident” = 4, “Completely unconfident” = 5; a value of 6 was attributed if PE signs were never used. A total score ranging from 9 to 54 could be obtained when all answers were added. Nine to 18 were considered as “High confidence”, 19 to 36 as “Intermediate confidence” and 37 to 54 as “Low confidence”. Finally, participants informed how frequent (1 = “Almost ever” to 3 = “Occasionally” to 5 = “Never”) they were using POCUS and they were referring for radiologist ultrasonography or chest X-ray. Answers were grouped in “Often” (i.e. “Almost ever” to “Often”) and “Seldom “ (i.e. “Occasionally” to “Never”). Characteristics of participants were collected and included, among others, place of practice (e.g. tertiary referral hospital), obtained or targeted speciality, years of clinical practice and previous POCUS training. The original survey is available online (in French).

The survey was hosted online by a commercial site (SurveyMonkey). A link to the survey was distributed by email to 197 GPs and to the heads of internal medicine (IM) and emergency medicine (EM) departments of hospitals in French-speaking cantons of Switzerland, who would subsequently distribute it to all doctors working in their facilities. The data were recorded anonymously; an email recall was planned, but failed to be sent because of Covid-19 pandemic and subsequent clinical priorities.

Descriptive statistics were used to present characteristics of participants and results. Proportions were compared with Pearson chi-square test. A two-sided P value of less than 0.05 was considered to infer statistical significance. Statistical analyses were performed using SPSS, version 26. Local ethical committee confirmed that a formal approval was unnecessary for the present study.

## Results

From November 2019 to January 2020, the survey was distributed to 197 GP and to the heads of IM and EM departments of 12 hospitals in French-speaking Switzerland, who relayed it to 667 in-hospital physicians (IHP). A total of 246 physicians answered to the survey (39 GP and 207 IHP) corresponding to a total response rate of 28.5%. Most participants (77.6%) came from the Geneva lake area, worked in hospitals (59% in tertiary-referral centres, 25% in secondary-care centres), and obtained or targeted a specialisation in internal medicine (IM) (93.1%). It is worth noting that in Switzerland IM title is required for GP and that emergency medicine (EM) is a complementary certificate, often obtained after an IM speciality. Characteristics of participants are presented in Table 1.

**Table 1:** Demographics and characteristics of study participants

Total participants, No. (%)	246 (100)
Region of work, No. (%)	
Geneva lake area	191 (77.6)
Others	55 (22.4)
Place of practice, No. (%)	
Tertiary-referral hospital	146 (59)
Secondary-care hospital	61 (25)
Primary care	39 (16)
Type of activity, No (%)	
Internal medicine	120 (50)
Emergency medicine	59 (24)
Family doctor	39 (16)
Others	24 (10)
Obtained/Targeted speciality, No. (%)	
General internal medicine	215 (93)
Anaesthesiology	8 (3.5)
Intensive care	8 (3.5)
Clinical position, No. (%)	
Resident	103 (41)
Chief resident	62 (25)
Attending physician	28 (11)
Chief of service	14 (6)
General practitioner	39 (16)
Years of practice, No. (%)	
< 5 years	104 (42.8)
5–15 years	89 (36.6)
> 15 years	50 (20.6)
Previous structured POCUS formation, No. (%)	
Yes	122 (50)
No	122 (50)

### Physical examination

In case of suspected abdominal or pleural free fluid, almost all respondents declared to look for PE signs of ascites and pleural effusion (90% and 88%, respectively). In-hospital physicians reported a more frequent use of PE in the last three months than did GP (70% vs 36% for pleural effusion, *P* = 0.001; 35% vs 5% for ascites, *P* = 0.001). When compared with EM doctors, IM physicians tended to report a more frequent use of PE (43.3% vs 24.6% for ascites, *P* = 0.015; 75.8% vs 69.5% for pleural effusion, *P* = 0.001) and a greater global confidence in PE (89.3% of high or intermediate confidence versus 84%, *P* = 0.001, Fig. 2). Most participants reported to be relatively confident in conventional signs of pleural effusion (91% for diminished breath sounds and 80% for dullness to percussion) and ascites (66% for flank dullness). However, more than half declared to have no confidence in most of the remaining signs. No significant difference was observed between regions of work for frequency of use and confidence. Table 2 reports proportions of confidence in PE as evaluated in our survey, along with sensitivity, specificity, positive, and negative likelihood ratios for each of PE signs as reported in previous systematic reviews and meta-analyses [10, 11].

**Table 2:** Rate of confidence sensitivity, specificity, and likelihood ratios for physical examination signs of abdominal and pleural free fluid

Physical examination signs	Fraction of confident, %	Diagnostic performances according to previous systematic reviews and meta-analyses [10, 11]			
		Sensitivity (95% CI)	Specificity (95% CI)	Positive LR (95% CI)	Negative LR (95% CI)
Abdominal free fluid					
Flank dullness	66	0.84 (0.68–1.00)	0.59 (0.47–0.71)	2.0 (1.5–2.9)	0.3 (0.1–0.7)
Fluid wave	52.6			6.0 (3.3–11.1)	
Shifting dullness	49.2	0.62 (0.47–0.77)	0.90 (0.84–9.6)		0.4 (0.3–0.6)
‘Glaçon’ sign	28	0.77 (0.60–0.88)	0.72 (0.63–0.81)	2.7 (1.9–3.9)-	0.3 (0.2–0.6)
Pleural free fluid					
Diminished breath sounds	90.6	0.42–0.88	0.83–0.90	4.3–5.2*	0.2–0.6*
Dullness to percussion	79.9	0.73 (0.61–0.82)	0.91 (0.88–0.93)	8.7 (2.2–33)	0.3 (0.1–3.3)
Reduced tactile vocal fremitus	31.6	0.82	0.86	5.7 (4.0–8.0)	0.2 (0.1–0.4)
Asymmetric chest expansion	25.2	0.74	0.91	8.1 (5.2–12.7)	0.3 (0.2–0.5)
Pleural friction rub	35.9	0.05	0.99	3.9 (0.8–18.7)	1.0 (0.9–1.0)

*CI* confidence interval, *LR  *likelihood ratio.

*No pooled analysis because the data was per lung region or hemithorax, not per patient

### Point-of-care ultrasonography

**Fig. 1:**
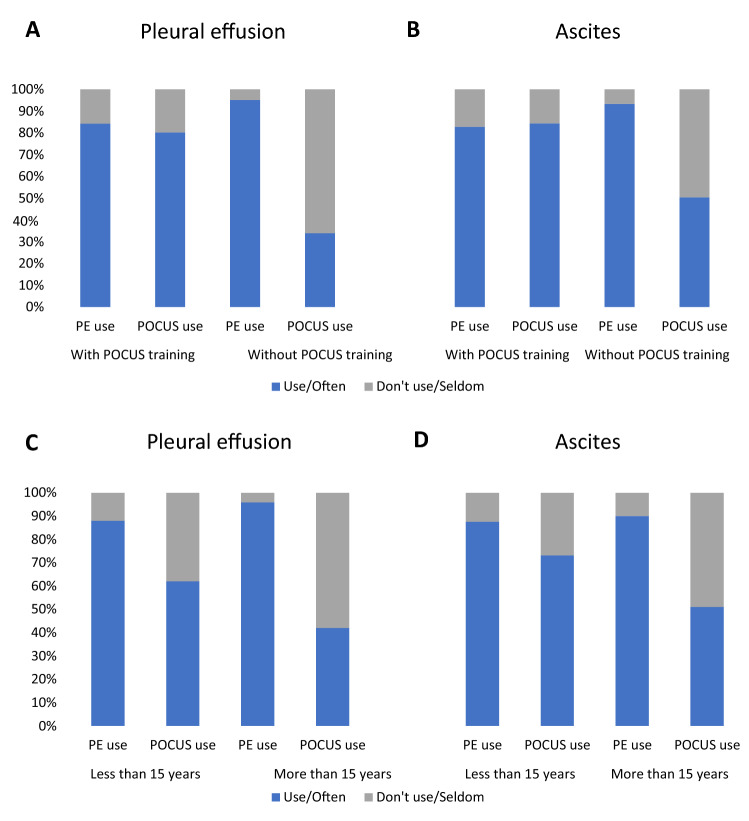
Physical examination and POCUS use according to previous POCUS training (Panel AB) and years of clinical experience (Panel CD)

**Fig. 2:**
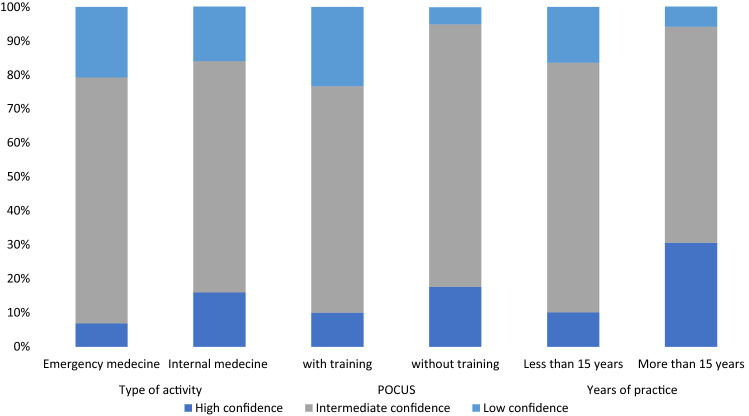
Global confidence in physical examination according to type of activity, previous POCUS training, and years of clinical experience

Globally, less than two-third of respondents affirmed to frequently use POCUS in case of suspected abdominal or pleural free fluid (57.1% for pleural effusion; 67.8% for ascites). Half of participants (50.0%) affirmed having attended a structured POCUS course. More than three quarters (75.9%) of participants reported to easily access to US devices at their workplace. Greater proportions of POCUS-trained physicians (55.1% versus 23.1%, *P* = 0.001) and greater devices availability (87.4% versus 15.4%, *P* = 0.001) were reported by IHP when compared with primary-care physicians. In hospitals, a larger proportion of EM physicians declared to be POCUS-trained than did IM physicians (77.6% versus 44.6%, *P* = 0.001). POCUS-trained physicians reported a lower use of PE than did untrained for both ascites (82.8% versus 93.4%, *P* = 0.01) and pleural effusion (84.3% versus 95.1%, *P* = 0.006). Global confidence in PE signs was also lower for POCUS-trained doctors (23.3% of unconfident versus 5% in untrained ones, *P* = 0.001). Moreover, POCUS-trained participants mentioned a greater POCUS use in case of suspected abdominal (84.4% versus 50.4% of frequent users, *P* < 0.001) or pleural free fluid (80.3% versus 33.9%, *P* < 0.001) and lower rate of radiologist referral for diagnosis of ascites (28.1% versus 71.3%, *P* < 0.001) and pleural effusion (21.3% versus 42.6%, *P* < 0.001). The results are presented in Fig. 1, Panel AB, and Fig. 2.

### Work experience

Globally, most of physicians with > 15 years of experience were confident in PE (94.0% of high or intermediate confidence), whereas less than half affirmed to use POCUS to confirm presence of ascites (49.0%) or pleural effusion (42.0%). Proportion of confident in PE was significantly lower (83.6. %, *P* = 0.001) and POCUS use greater (73.0% and 62.0% respectively, *P* = 0.001) in less experienced doctors. As a result, senior doctors declared referring more frequently to radiologist for diagnosis (77.0% versus 44.0% for ascites, 46.0% versus 28.0% for pleural effusion, *P* = 0.012). The results are presented in Fig. 1, Panel CD, and Fig. 2.

## Discussion

We observed that PE is frequently performed for diagnosis of ascites and pleural effusion by both ambulatory and in-hospital physicians. If most of them reported to be confident in component of conventional PE (abdominal flank dullness, diminished breath sounds, and dullness to thorax percussion), rates of confidence were significantly lower for the other clinical signs. Interestingly, there was no apparent relationship between reported confidence and diagnostic performances, with lower confidence given to signs with better likelihood ratios (e.g., reduced tactile vocal fremitus and asymmetric chest expansion, see Table 2). Only 62.0% of participants declared a regular use of POCUS, despite growing evidence of superiority over PE regarding the detection of free fluids [27]. POCUS use tends to be greater in workplaces with higher US devices availability, in younger and in POCUS-trained physicians. In contrast, senior clinicians tend to be more confident in PE, to have a minor employ of POCUS and refer more to radiologists for diagnosis. In a previous study, 2864 physicians were surveyed on the value of PE with questions on the usefulness and frequency of use of 58 PE signs for a large variety of diagnoses [25]. For ascites and pleural effusion, participants were surveyed on two PE signs. Abdominal percussion for ascites and chest percussion for abnormal dullness were deemed useful by 90% and 91% of respondents, respectively, while their reported frequency of use was lower, around 70%. In the present study, we surveyed physicians on their perceived confidence in nine PE signs; thus, focusing the investigation on the clinical approach to patients with suspected ascites or pleural effusion. Although not directly comparable with the usefulness and frequency of use, our results on the perceived confidence in the above mentioned two main PE signs are similar to those of this previous survey. Importantly, our survey extends these findings by providing insight into various interactions between the perceived confidence in PE signs and practice of POCUS, clinical experience, and clinical setting.

Almost 30 years after the advent of POCUS [28], this valuable tool is yet incompletely integrated in clinical practice. Resistance within the medical community in adoption of innovations is a well-known phenomenon [29] particularly affecting senior physicians. Other barriers to POCUS implementation have been previously identified and include insufficient training time, unavailability of trainers, absence of structured curricula, material and financial support, as well as lack of consideration by US specialist [6, 30–32]. In Switzerland, particularly in the French-speaking part, these barriers have been predominant in the early 2000s and are now slowly overcame due to the implication of a growing population of POCUS leaders.

POCUS was initially developed in intensive care and EM; it is much more integrated in clinical practice in these specialities when compared with IM, being on the contrary a late POCUS-adopter discipline [18]. Furthermore, mastering of extended focused assessment with sonography for trauma (eFAST) is required for the complementary certification in EM, whereas no specific POCUS competence is yet necessary for obtaining IM title in Switzerland. For these reasons, we observed a greater proportion of EM physicians having attended a structured course and using POCUS compared with IM IHP or GP. Interestingly, in our survey lower use and confidence in PE was reported by participants affirming a greater POCUS use. This may be due to POCUS better diagnostic accuracy or may be related to the higher trust given by new generations of physicians to technologies or to their ability in integrating innovations. Globally, these results suggest that a shift of practice toward integration of POCUS has started and may become evident in next decades.

The present study has several limitations. First, less than one third of contacted physicians answered to the survey. Although low, this is usual in such professional surveys [33] and slightly greater than the answer rate obtained in the previously reported international survey (24%) [25]. Second, the use of PE for ascites and pleural effusion was asked with two general questions, preventing us to conclude about the frequency of use of each previously described PE signs. Indeed, regarding these specific signs, only their attributed confidence was reported. Some of these signs being detected by routine PE (e.g. bilateral lung auscultation and abdominal percussion), frequency of use of PE in last 3 months could be overestimated in IHP, due to the custom of performing a baseline complete PE in all hospitalized patients. Finally, the survey was limited to the French-speaking cantons of Switzerland and conclusion may not be generalizable to other regions or countries. In fact, POCUS is much more integrated in German-speaking part of Switzerland, as suggested by a recent cross-sectional survey among GP, reporting a frequent POCUS use for half of participants [34], as compared to only 15.4% of GP included in our survey. This is unlikely to lessen the differences observed in the present survey, but should rather strengthen them.

In conclusion, PE still occupies a central place in detection of free fluids. The established POCUS superiority over PE signs for fluid detection, however, has introduced a progressive change of practice that may become evident in the following generations of physicians. As it happened with the revolutionary advent of stethoscope 200 years ago, POCUS should be progressively integrated and enhance physical examination, instead of replacing it. For this reason, teaching efforts should be placed in valuable components of PE and in developing structured POCUS training.

## Data Availability

Our data are accessible to researchers upon reasonable request for data sharing to the corresponding author.

## Appendix 1

Study questionary (French).
